# The Relation between Media Consumption and Misinformation at the Outset of the SARS-CoV-2 Pandemic in the US

**DOI:** 10.37016/mr-2020-012

**Published:** 2020-04-20

**Authors:** Kathleen Hall Jamieson, Dolores Albarracin

**Affiliations:** (1) Annenberg Public Policy Center, University of Pennsylvania,; (2) Department of Psychology and Gies Business School, University of Illinois at Urbana Champaign

## Abstract

A US national probability-based survey during the early days of the SARS-CoV-2 spread in the US showed that, above and beyond respondents’ political party, mainstream broadcast media use (e.g., NBC News) correlated with accurate information about the disease’s lethality, and mainstream print media use (e.g., the New York Times) correlated with accurate beliefs about protection from infection. In addition, conservative media use (e.g., Fox News) correlated with conspiracy theories including believing that some in the CDC were exaggerating the seriousness of the virus to undermine the presidency of Donald Trump. Five recommendations are made to improve public understanding of SARS-CoV-2.

## Essay summary

With coverage of SARS-CoV-2 dominating discussions on air, in print, and online, between March 3 and March 8, 2020 we fielded a US national probability phone survey of 1,008 respondents to (a) determine the accuracy of the public’s understanding of the relative lethality of the seasonal flu and the coronavirus and of the need to prevent SARS-CoV-2’s spread by hand washing and avoiding those showing symptoms of respiratory illness, and (b) assess the association between use of various media channels and accurate and inaccurate beliefs and conspiracy theories about SARS-CoV-2 while controlling for potential differences between Republicans and Democrats, who have been reported to differ in concern with SARS-CoV-2 (Gallup, 2020).

## Implications

Public understanding of needed preventative measures and rejection of bogus ones is important because SARS-CoV-2 is highly contagious and potentially lethal (cdc.gov). Pollsters have identified partisan differences in views on SARS-CoV-2. In particular, a number of March 2020 polls showed that Republicans were less worried than were Democrats about exposure to the virus (Gallup 2020), less likely to consider the SARS-CoV-2 outbreak a major health threat (Pew 2020), and more likely to approve of President Donald Trump’s handling of the “coronavirus pandemic” (Marist, 2020). Like this work, our early March data registered differences tied to partisanship in their concern about SARS-CoV-2, specifically that Republicans were less knowledgeable about the relative lethality of SARS-CoV-2. In addition, our data suggested an association between exposure to some kinds of media, conservative and social media in particular, and being misinformed, associations that persist when partisanship is considered. Our data warrant five recommendations.

### The need for proactive communication about prevention

Because hand washing and social distancing can prevent the spread of respiratory viruses including the flu, the finding that early in March, 87% believed that these practices were preventative signals a success of public health messaging. However, the gaps in the public’s background knowledge that we identified should alert public health officials to the ongoing need for effective communication of needed information long before a crisis.

Several areas need attention. First, the finding that 21% thought that taking vitamin C probably or definitely prevents infection and 26% were unsure of whether it would or not suggests unwarranted public confidence in this supplement. As a Cochrane meta-analysis confirmed (Cochrane 2013), vitamin C consumption does not even prevent the common cold “in the ordinary population,” contrary to what the commonplace claim avers. Nor, despite the claims on social media sites, does it prevent Anthrax and crib death (Kata 2010). Like those other false claims, the one asserting that taking vitamin C prevents one from contracting SARS-CoV-2 was circulating on Facebook in January 2020 (BBC Monitoring & UGC Newsgathering, 2020).

### Find out what misinformation to debunk

Because debunking misinformation including conspiracy theories is difficult (Chan et al., 2017), and not without potential unintended consequences (Nyhan et al., 2014), before deciding whether to debunk a conspiracy theory or other misinformation, fact-checking organizations need to know that enough people have embraced it to be worrisome. In the absence of such prevalence data, corrective efforts may do more harm than good by inadvertently increasing awareness of the problematic claim. One possible benchmark is to correct for beliefs considered salient in a population, which according to Ajzen and Fishbein (1980) is at least 10% of a population.

The individual conspiracy theories we studied met or passed this threshold. Ten percent of our survey respondents characterized as probably or definitely true the conspiracy theory that the US government created the virus, a conclusion that calls into question the integrity of the US government at a time at which public confidence is required to mount a national defense against a spreading menace. Among the sources circulating this canard were high-level Chinese officials who claimed that it was the US military that brought the virus to China (Reuters, 2020).

Nearly one in five of our respondents (19%) reported believing that some in the CDC are exaggerating the seriousness of the virus to undermine the Trump presidency^2^. This assumption has the potential to engender distrust in one of the two US government agencies tasked not only with protecting public health but also with communicating accurate information about ways to protect oneself and others. On social media, this theory was advanced under headlines such as “Coincidence? CDC Official Hitting the Coronavirus Panic Switch is Rod Rosenstein’s Sister” (O’Hara, 2020). Rosenstein is a former deputy attorney general who played a central role in the Mueller investigation of Russian interference in the 2016 US presidential election.

The notion that the virus was created by the Chinese as a bioweapon, which has the potential to fuel xenophobia and racism, was rated “probably true” or “definitely true” by 23% of our survey respondents. This theory was floated by Senator Tom Cotton (R-AR) on Fox News in mid-February, endorsed by Steve Bannon, former advisor to President Donald Trump (Stevenson, 2020), peddled in the conservative Washington Times (Gertz, 2020), and touted by conservative talk radio host Rush Limbaugh who said, “It probably is a ChiCom laboratory experiment that is in the process of being weaponized” (Limbaugh, 2020). Our data suggest that it makes more sense for fact-checkers to take on the CDC and Chinese bioweapon claims than the one alleging that the virus was created by the US.

### A baseline for monitoring social media interventions

By offering an early window on the level of public information and belief in conspiracy theories about SARS-CoV-2, this study provides a baseline that one can use to assess the success of the social media platforms’ efforts to blunt misinformation. As this study was fielding on March 3rd, Facebook’s CEO Mark Zuckerberg announced that “Facebook was removing false claims and conspiracy theories flagged by global health organizations and the company is blocking people from running ads that try to exploit the fears of the public by pitching snake oil cures” (Techcrunch, 2020). Moreover, Twitter, YouTube, and Facebook now direct those searching for “coronavirus” to sources such as the Centers for Disease Control and Prevention (CDC). Twitter also initiated a campaign called #KnowTheFacts (Brandon, 2020). Two of the mistaken claims on which we focused have been interdicted by the platforms. Yet, before YouTube removed a video asserting that the pandemic had been bioengineered, 570,000 subscribers to the website SGT Report had potentially been exposed to it (Herrera, 2020). To the best of our knowledge, our study is the first to assess public belief in the conspiracy theories and preventive effects of vitamin C that circulated on social media.

### Proposed interventions in conservative media

The data in this study should motivate public health officials to place public service announcements, encourage hyperlinks to the CDC information pages, and seek interviews on outlets whose audiences are less knowledgeable, more misinformed, or more accepting of conspiracy theories. This strategy was exemplified by National Institute of Allergy and Infectious Diseases Director Dr. Anthony Fauci, who on March 11th on Fox News responded to Sean Hannity’s request to compare the seasonal flu to the coronavirus by noting, “The mortality for seasonal flu is 0.1 [percent]” and the coronavirus is “10 times more lethal than the seasonal flu. You gotta make sure that people understand that!” (Fox News, 2020). Importantly, in that interview on Hannity’s top-rated Fox program, the host repeatedly vouched for Fauci’s credibility.

Among the reasons that credible sources should place such information in conservative media venues is that conservative talk radio listeners and Fox viewers tend to be older, and as such part of the group most susceptible to SARS-CoV-2 complications (cdc.gov). Fauci’s statement directly rebutted a canard that had been trafficked in conservative media where Rush Limbaugh said, “I’m dead right on this. The coronavirus is the common cold, folks” (Limbaugh, February 24 2020), and that “The fatality rate of this virus is less than the flu, far less than the flu. But look at how it’s been hyped” (Limbaugh, February 25, 2020). Furthermore, “medical contributor” Dr. Mark Siegel stated on Sean Hannity’s top-rated Fox program, “the virus should be compared to the flu. Because at worst, at worst, worst case scenario it could be the flu” (Fox News, March 6, 2020).

### Newspapers: Take down paywalls on SARS-CoV-2 coverage

Our finding that reading mainstream print is associated with higher levels of knowledge should incentivize newspapers to follow the lead of outlets such as the Washington Post and New York Times and eliminate the paywall on their coronavirus coverage. Readers who appreciate this contribution to public health might respond by subscribing or, in the case of the Guardian, which does not have a paywall, by donating to that organization.

## Findings

**Figure 1.**
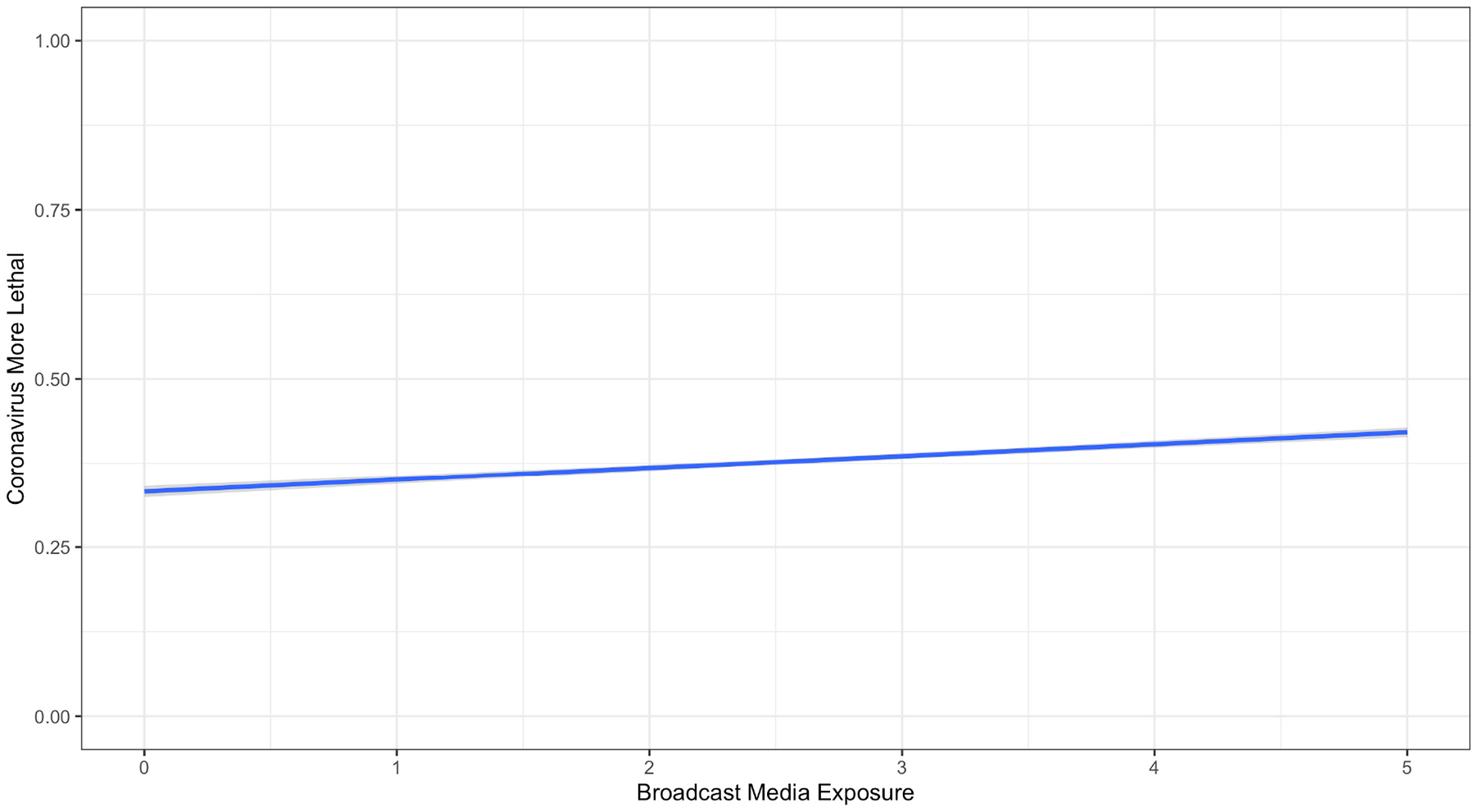
Association between Mainstream Broadcast Media Exposure and Perceived Lethality of SARS-CoV-2

**Figure 2.**
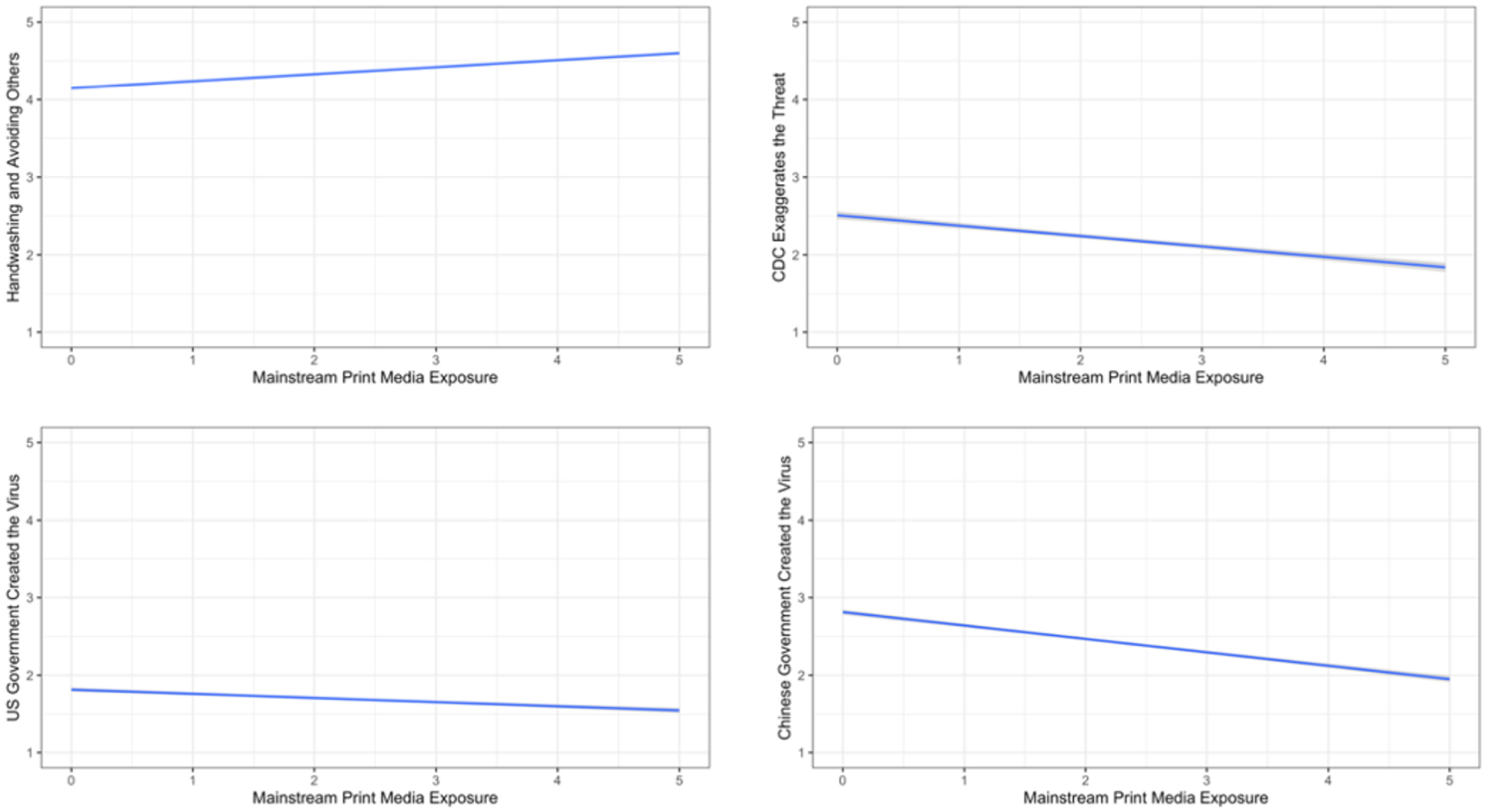
Associations between Mainstream Print Media Exposure and Information/Misinformation

**Figure 3.**
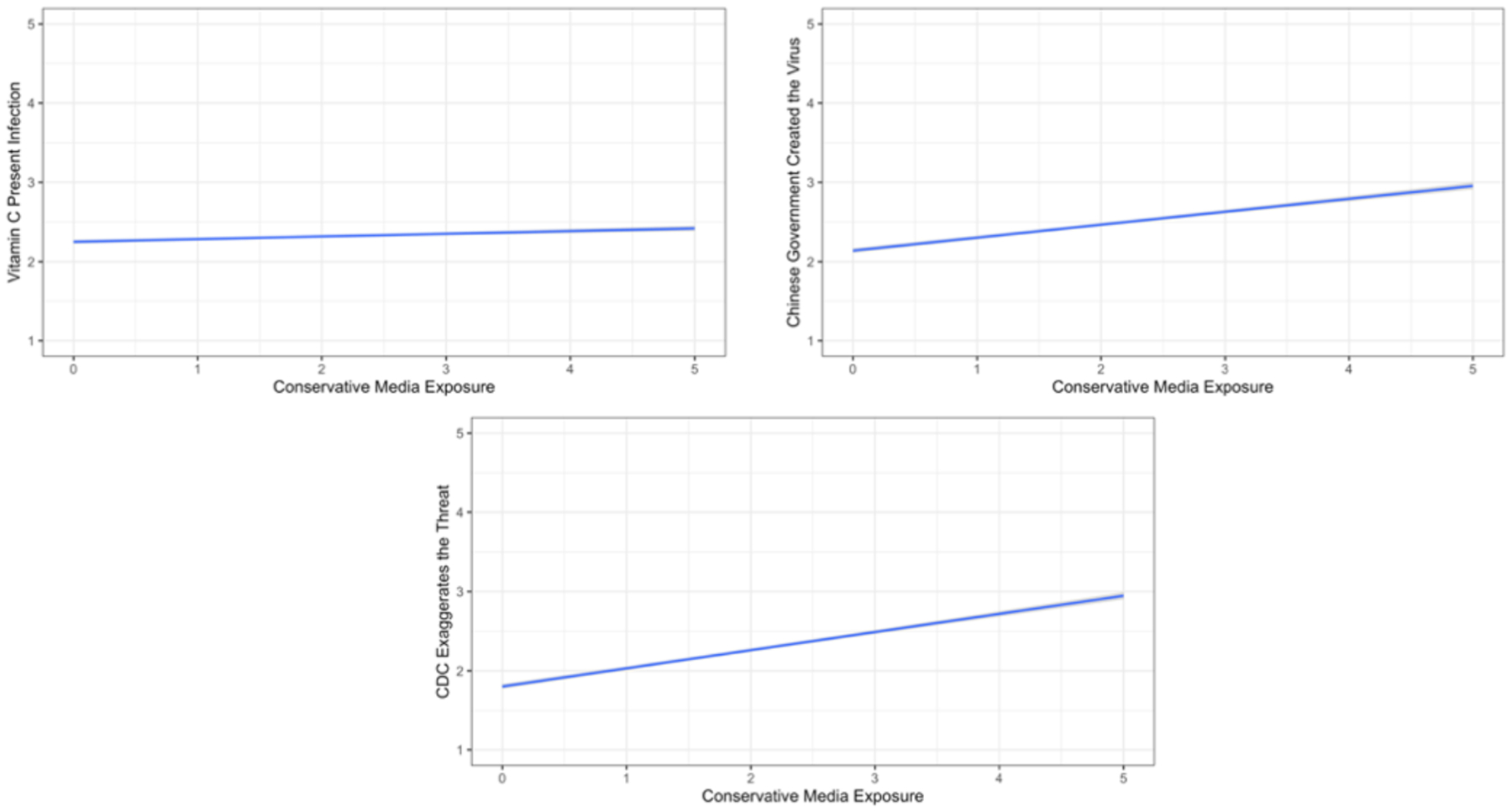
Associations between Conservative Print Media Exposure and Misinformation

**Figure 4.**
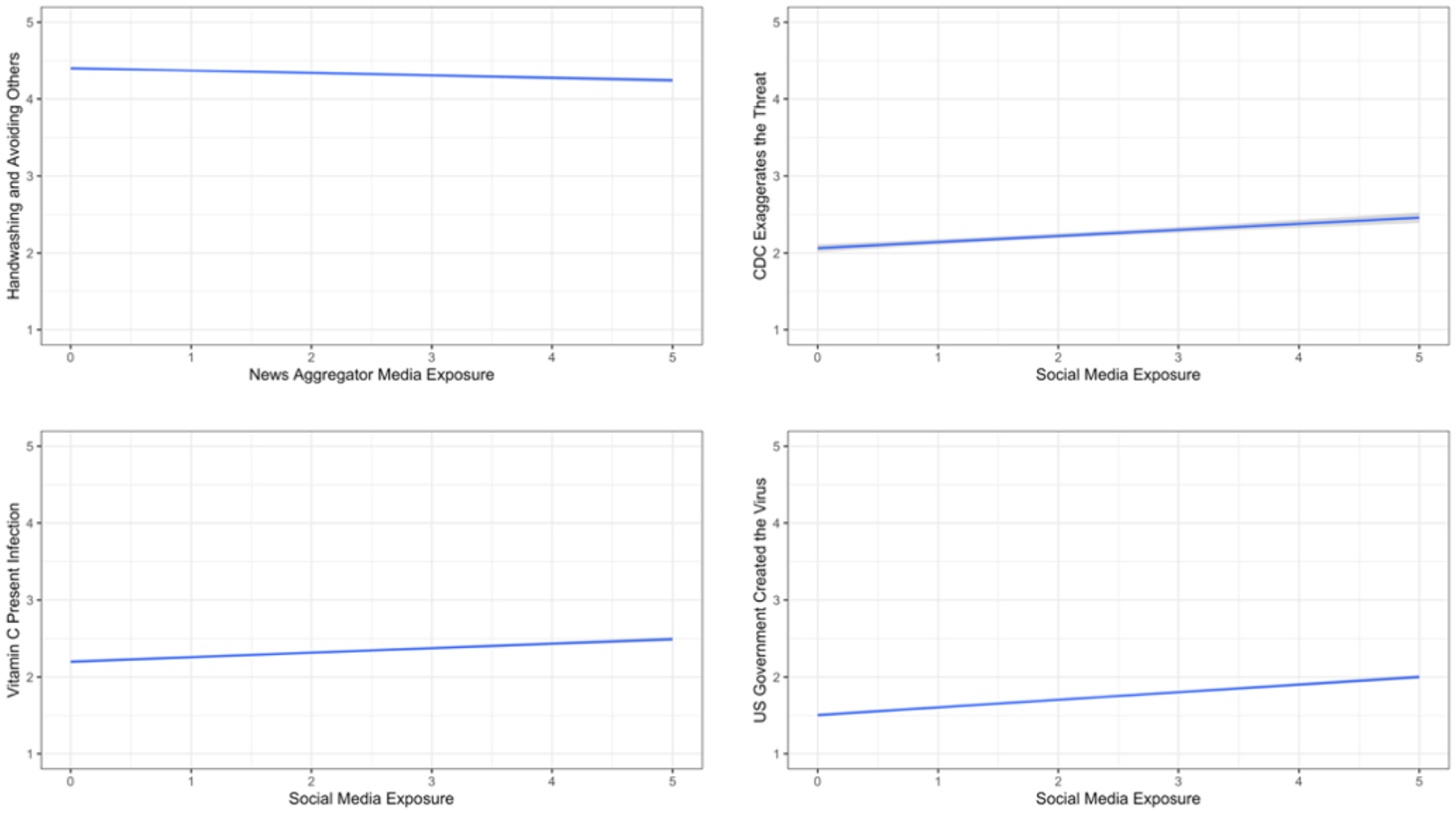
Associations between Yahoo or Google News Aggregators or Social Media Exposure and Information/Misinformation3.

The last panel of Table 1 presents the means and standard deviations for exposure to different sources of information. Table 2 presents the associations of respondents’ beliefs in the information/misinformation of interest with media exposure. All associations stem from a multiple-regression analysis with controls for political party, political ideology, education, gender, and age. Figures 1–4 present significant regression lines corresponding to the significant media predictors in Table 2. All simple correlations appear in the Appendix and indicate relations among party, ideology, demographic, and media predictors and hence the need to control for them through multiple regressions.

### Familiarity with SARS-CoV-2

Familiarity with the novel coronavirus was high. Ninety-six percent of the sample reported having heard about it.

#### A. Level of Information: Low Levels of Information about Lethality and Prevention and High Levels of Misinformation

- The public’s sense of the relative risks of death from the coronavirus as opposed to the flu was wanting. Although 39% knew that a person with coronavirus was more likely to die as a result than was a person who had contracted the seasonal flu, 38% thought that one disease was as likely as the other to result in death, 13% considered the seasonal flu more deadly, and 8% endorsed “it depends” (see Table 1).
- There were gaps in information about the need for hand washing and avoiding close contact with those showing respiratory symptoms (the concept of social distancing was not yet prevalent in the national dialogue), as well as misinformation that taking vitamin C is preventative (see Table 1). Specifically, 13% believed that it was probably or definitely false or were unsure whether hand washing and avoiding contact with symptomatic people prevent infection. Moreover, 21% reported that it is definitely or probably true that taking vitamin C can prevent a person from being infected with coronavirus (see Table 1). An additional twenty six percent were unsure.

#### B. Partisanship: Democrats and Republicans Differed in Perceived SARS-CoV-2 Lethality

- Democrats were more likely than Republicans to know that the coronavirus is more lethal than the flu (see Table 2).
- Republicans also were more likely to believe that the CDC was exaggerating the threat of the coronavirus to hurt President Donald Trump (see Table 2).

#### C. Associations between Media Exposure and Information/Misinformation While Taking Ideology and Party into Account

##### 1C. Mainstream Broadcast and Print Media Exposure Correlates with More Information and Less Misinformation Even after Taking Ideology and Party into Account

- Exposure to mainstream broadcast and cable correlated positively with reporting that the novel coronavirus is more lethal than the flu (for a similar mainstream media effect, see Stecula, Kuru, & Jamieson, 2020) (see Table 2 and Figure 1).
- Exposure to mainstream print was positively associated with holding more accurate beliefs about prevention of infection with SARS-CoV-2. Specifically, exposure to sources such as the Associated Press, The New York Times, the Washington Post, or the Wall Street Journal was positively associated with accurately believing that regular hand washing and avoiding contact with symptomatic people prevent infection (see Table 2 and Figure 2).
- Exposure to mainstream print was negatively associated with the beliefs that taking vitamin C can prevent infection, some in the CDC were exaggerating the threat to harm Trump, and the virus is a bioweapon created by the Chinese government (see Table 2 and Figure 2).

##### 2C. Conservative Media Exposure Correlates with Higher Levels of Misinformation

- Use of conservative media (sources such as Fox News and Rush Limbaugh) correlated with beliefs in the malign underlying motives of some at the CDC and the Chinese origin of the virus (see Table 2 and Figure 3).
- Furthermore, exposure to conservative media correlated with unwarranted confidence in vitamin C consumption as a means of preventing infection by SARS-CoV-2 (see Table 2 and Figure 3).

##### 3C. Social Media Exposure Correlates with Lower Levels of Information and Higher Levels of Misinformation

- Exposure to outlets such as the web aggregators Google News and Yahoo News correlated with lower belief in the efficacy of regular hand washing and avoiding contact with symptomatic individuals (see Table 2 and Figure 4).
- Exposure to sources such as Facebook, Twitter or YouTube was positively correlated with belief in the efficacy of vitamin C, the belief that the CDC was exaggerating the threat to harm President Trump, and the belief that the virus was created by the US government (see Table 2 and Figure 4).

## Methods

The survey was conducted for the Annenberg Public Policy Center at the University of Pennsylvania via telephone by Social Science Research Solutions (SSRS), an independent research company. Interviews were conducted with a sample of **1,008** respondents between March 3 and March 8, 2020. Of the total sample, 701 participants were surveyed by cell phone, and the remaining via landlines. Although the majority of the respondents answered in English, 35 participants completed the survey in Spanish. The margin of error for total respondents is +/−3.57% at the 95% confidence level. Response rate was 3.5%. More information about SSRS can be obtained by visiting www.ssrs.com.

The survey items were developed after extensive pretesting of both the media and the belief measures. First, pilot data conducted during 2019 indicated that the grouping of conservative news outlets was relatively homogeneous in capturing demographically similar audiences. These measures were formally validated by Jamieson and Hilgard (2017). Second, an online pilot survey conducted by SSRS in February 2020 pilot tested four of the belief measures (that hand washing and avoidance of contact with symptomatic others prevented infection; that the virus was created by the Chinese government; that the virus was created by the US government; and that vitamin C prevented infection), which correlated strongly with other conspiracy theories (i.e., Agenda 21, the link between MMR and autism, and the belief that Obama was not born in the US). These pilot data thus provided indication of the construct validity of our belief measures. In addition, the February pilot data showed that the media measures predicted beliefs in theories described in conservative and social media outlets.

The survey first asked whether the respondent had “read, heard, or seen anything about a virus called the coronavirus, also known as COVID-19, first detected in Wuhan, China in December 2019,” with the options being “Yes” or “No.” To assess information about the risk of coronavirus compared to the seasonal flu, we asked “If someone gets the seasonal flu and another gets the coronavirus, which person do you think is more likely to die from the disease?” Three response options were offered: “The person with seasonal flu”; “the person with coronavirus”; “they are equally likely to die of the disease they have”; “it depends”; and “I don’t know.”

In addition, of interest to this study were five items assessing respondents’ beliefs that: (a) “the ways to prevent infection with the coronavirus include regular hand washing and avoiding those showing symptoms of respiratory illness”; (b) “taking vitamin C can prevent a person from being infected with the coronavirus”; (c) “some in the U.S. Centers for Disease Control and Prevention, also known as the CDC, are exaggerating the danger posed by the coronavirus in order to damage the Trump presidency”; (d) “the U.S. government created the coronavirus”; and (e) “the coronavirus was created by the Chinese government as a biological weapon.” Participants were read a statement, after which the interviewer asked, “Do you believe this is…?”. Participants received the following options: 1. “Definitely true,” 2. “Probably true,” 3. “Probably false,” 4. “Definitely false,” 8: “Not sure.” Refusals were coded as 9 and scores were reversed so that higher values indicate more agreement: 1 indicated “definitely false” and 5 indicated “definitely true.” “Not Sure” (8) was recoded 3 to reflect the middle point^3^.

We also measured sources of information. Specifically, on a scale from 0 (*no information*) to 5 (*a lot of information*), participants were asked to report how much information they receive from sources such as: (a) ”Associated Press, The New York Times, the Washington Post, or the Wall Street Journal,” which we consider mainstream print outlets; (b) “Fox News, Rush Limbaugh, Breitbart News, One America News, or The Drudge Report,” which were considered conservative outlets; (c) “MSNBC, Bill Maher, or Huffington Post,” which we treat as liberal sources; (d) “ABC News, CBS News, or NBC News,” which were considered mainstream broadcast; (e) “Google News or Yahoo News,” which were considered social media news aggregators; and (f) “Facebook, Twitter, or YouTube,” which were considered social media sources.

Table 3 describes the sample, and shows not only that it is similar to the US population in sex, age, race, ethnicity, and education, but also that it had similar percentages of self-reported conservatives, moderates, and liberals.

## Supplementary Material

1

## Figures and Tables

**Table 1.** Information, Misinformation, and Media Use.

Items	Statistics	
	Frequency	%
Reports that the novel coronavirus is more lethal than the flu	390	39
Do you believe this is…?		
Regular hand washing and avoiding people with symptoms		
Definitely false	26	3
Probably false	29	3
Not sure	74	7
Probably true	364	36
Definitely true	512	51
Taking vitamin C		
Definitely false	295	30
Probably false	235	23
Not sure	260	26
Probably true	194	19
Definitely true	18	2
The CDC exaggerate the danger posed by the virus to hurt Trump		
Definitely false	370	37
Probably false	242	24
Not sure	201	20
Probably true	128	13
Definitely true	59	6
The U.S. government created the virus		
Definitely false	547	55
Probably false	222	22
Not sure	134	13
Probably true	77	8
Definitely true	21	2
The Chinese government created the virus		
Definitely false	260	26
Probably false	272	27
Not sure	247	25
Probably true	181	18
Definitely true	47	5
Do you believe this is…? (1: definitely false to 5: definitely true)		
	M	SD
Regular hand washing and avoiding people with symptoms	*4.30*	*0.92*
Taking vitamin C	2.41	1.15
The CDC exaggerate the danger posed by the virus to hurt Trump	2.27	1.24
The U.S. government created the virus	1.80	1.07
The Chinese government created the virus	2.49	1.19
How much information do you get from the following sources? (0: a bit to 5: a lot)		
	M	SD
Mainstream Print Media (Associated Press, the New York Times, the Washington Post, or the Wall Street Journal)	2.16	1.76
Conservative Media (Fox News, Rush Limbaugh, Breitbart News, One, America News, or The Drudge Report)	1.74	1.82
Mainstream Broadcast Media (ABC News, CBS News, or NBC News)	2.72	1.72
Liberal Media (MSNBC, Bill Maher, or Huffington Post)	1.62	1.68
Online News Aggregators (Google News or Yahoo News)	1.90	1.72
Social Media (Facebook, Twitter, or YouTube)	2.19	1.84

Results are weighted to approximate the US population.

**Table 2.** Predicting Beliefs from Sources of Information.

	Corona is more lethal than the flu^1^		Beliefs				
Predictors	Logistic Regression	Linear Regression	Regular hand washing and avoiding people with symptoms	Taking vitamin C	The CDC exaggerate the danger	The US government created the virus	The Chinese government created the virus
Non-media variables							
Political party	−0.20*	−0.10*	0.05	−0.08	0.15***	−0.02	0.05
Conservative political views	0.07	0.06	−0.02	−0.03	0.08*	−0.05	0.05
Education	0.02	0.03	0.12***	−0.10**	−0.11***	−0.13***	−0.10**
Age	−0.01	−0.03	0.01	−0.12**	0	−0.08*	0.04
Female sex	−0.47***	−0.10**	0	0.10**	−0.03	0.04	0.03
Media variables							
Mainstream Print Media (Associated Press, the New York Times, the Washington Post, or the Wall Street Journal)	−0.03	−0.02	0.16***	−0.03	−0.09*	−0.09*	−0.18***
Conservative Media (Fox News, Rush Limbaugh, Breitbart News, One, America News, or The Drudge Report)	−0.02	−0.01	−0.02	0.10**	0.21***	0.01	0.17***
Mainstream Broadcast Media (ABC News, CBS News, or NBC News)	0.10*	0.09**	0.01	0.06	−0.05	−0.07	−0.03
Liberal Media (MSNBC, Bill Maher, or Huffington Post)	−0.06	−0.04	0.02	−0.04	0	0.02	−0.03
Online News Aggregators (Google News or Yahoo News)	0	0	−0.10**	0.06	0.02	0.05	0.02
Social Media (Facebook, Twitter, or YouTube)	0.03	0.04	0.03	−0.01	0.10**	0.11**	0.11**
*R* ^*2*^	.08	0.02	.05	.05	.18	.07	.14
*N*	953	953	950	949	947	948	953

*Note*.

*: *p* < .05,

**: *p* < .01.

***: *p* < .001.

Political party and ideology are scored so that higher numbers represent more conservative choices. Party: −1: democrat, 0: independent, 1: republican. Political ideology: 1(*very liberal*) to 5 (*very conservative*).

1 Responses as to whether corona is more deadly than the flu resulted in a dichotomous variable: 1: chose that it is more deadly, 0: did not choose that it is more deadly. Hence this variable was analyzed with both linear and logistic regressions. For the logistic regression, the Cox & Snell R_2_ is reported, along with unstandardized beta weights for the predictors. For linear regressions, coefficients for individual variables are standardized. Results are weighted to approximate the US population.

**Table 3.** Description of the Sample

	Frequency	%
Sex		
Female	488	48
Male	520	52
Age		
18–19	41	4
20–29	170	17
30–39	167	17
40–49	159	16
50–59	156	16
60–69	155	15
70–79	98	10
80+	62	6
Race/Ethnicity		
White Non-Hispanic	625	62
Black Non-Hispanic	116	12
Asian	26	3
Native American/American Indian/Alaskan Native	23	2
Native Hawaiian and Pacific Islander	5	1
White Hispanic	96	10
Black Hispanic	17	2
Unspecified Hispanic	46	5
Mixed	29	3
Refused	24	2
Education		
Less than high school graduate	81	8
High school graduate	304	31
Some college or associate degree	276	28
College	232	23
Postgraduate	103	10
Political party		
Republican	362	36
Independent	126	13
Democrat	520	52
Political views		
Very conservative	143	15
Somewhat conservative	199	21
Moderate	342	35
Somewhat liberal	174	18
Very liberal	113	12

Results are weighted to approximate the US population.

## Research questions

- In early March 2020, how informed was the US public about ways in which one can protect oneself from SARS-CoV-2 and of its relative lethality compared to the seasonal flu?
- Did party identification correlate with levels of information about the lethality of the virus?
- Does use of different types of media (e.g., mainstream, conservative, liberal; traditional or social media) correlate with accurate or inaccurate information regarding SARS-CoV-2 prevention? For example: Does use of any type of media correlate with information about lethality and appropriate methods of prevention? Does the use of social media rather than either broadcast or traditional print media covary with belief in misinformation and conspiracy theories regarding SARS-CoV-2? Does use of social media or of conservative media correlate with increased belief in conspiracy theories being trafficked in these venues?

## Footnotes

2 All statistics appear in Table 1, including means and standard deviations for these beliefs. However, given a benchmark of 10% for beliefs to be salient in a population (Ajzen & Fishbein, 1980), the percent of people who subscribe to misinformation is important.

3 5% of participants who chose “probably true” or “definitely true” in response to both the item stating that the Chinese government created the virus and that the US government created the virus. As shown in the Appendix, the correlation between these two beliefs was r = .33.

## Appendix Correlation Matrix

**Table:**

		2	3	4	5	6	7	8	9	10	11	12	13	14	15	16
**1. Corona more lethal**	1															
**2. Hand washing and avoiding**	−.076*	1														
**3. Taking vitamin C**	0	−.070*	1													
**4. The CDC exaggerate**	−.093**	−.044	.120**	1												
**5. US government**	.027	−.205**	.240**	.175**	1											
**6. Chinese government**	.037	−.117**	.162**	.339**	.331**	1										
**7. Political party**	−.063*	−.009	−.071*	.283**	−.015	.190**	1									
**8. Conservative political views**	.064*	.004	−.094**	.081*	.031	.070*	.218**	1								
**9. Education**	.010	.049	.009	−.009	−.026	−.025	.013	.073*	1							
**10. Age**	−.036	.008	−.112**	.018	−.144**	.037	.129**	.084**	.052	1						
**11. Female sex**	−.075*	.016	.092**	−.013	.031	.006	−.149**	−.049	−.007	.041	1					
**12. Mainstream media**	.007	.161**	−.001	−.158**	−.087**	−.216**	−.225**	−.059	.070*	−.106**	.028	1				
**13. Conservative media**	−.013	−.029	.062	.292**	.01	.231**	.352**	.135**	.054	.194**	−.016	−.026	1			
**14. Mainstream broadcast**	.05	.043	.041	−.102**	−.091**	−.087**	−.196**	−.137**	.101**	.198**	.098**	.312**	.070*	1		
**15. Liberal media**	−.002	.058	.022	−.084**	.018	−.090**	−.239**	−.036	.098**	.034	.075*	.474**	.079*	.395**	1	
**16. Google or Yahoo News**	.037	−.045	.102**	.073*	.111**	.05	−.031	−.102**	−.047	−.208**	.013	.212**	.124**	.068*	.246**	1
**17. Social media**	.053	−.012	.102**	.126**	.152**	.102**	−.054	−.021	0	−.391**	.082**	.151**	.079*	−.026	.127**	.483**

*: *p* < .05.

**: *p* <.01.

For ease of comparability, reports that the novel coronavirus is more lethal than the flu, which is a dummy variable, was analyzed using Pearson correlations as well. However, like in Table 2, logistic regressions led to the same variables being statistically significant and the same pattern of findings. Results are weighted to approximate US population.
