# Supplementary material for: The Relation between Media Consumption and Misinformation at the Outset of the SARS-CoV-2 Pandemic in the US
Source: Harv Kennedy Sch Misinformation Rev. Author manuscript; Available in PMC 2025 Sep 12. (PMC12424044; doi:10.37016/mr-2020-012)
Supplement: 1 [file NIHMS2109036-supplement-1.pdf]

4/27/20 8:37:00 PM
coronovirusqn.doc
Job #T8809
March 3, 2020

CORONAVIRUS

RECORD ALL ROTATE VARIABLES

Now I have some questions for you for a research study on issues related to health on behalf of the […]. You have been selected as part of a cross-section of American adults. I want to remind you that your participation in this study is completely voluntary and all responses will remain confidential in a password protected file in the […]. Your individual responses will never be released as findings will be reported in the aggregate. All data we collect will be de-identified, and could be stored and distributed for future research. The information that we gather may help us to provide invaluable data on a variety of health issues.

If there is a question you would rather not answer, then we will move on.

(IF NECESSARY) If you have any questions regarding this research or with regard to your participation, you can contact [….]

CORO1. Have you read, heard, or seen anything about a virus called the coronavirus, also known as COVID-19, first detected in Wuhan (WOO-han) China in December 2019?

1 Yes
2 No
8 (DO NOT READ) Don't know
9 (DO NOT READ) Refused

I'm going to read you some statements. For each one, please tell me if you believe it is true, false or if you aren't sure.

(ROTATE CORO2 AND CORO4)

COROQ2. The US government created the coronavirus.
Do you believe this is (READ LIST)?

(ROTATE 1-4/4-1 IN THE SAME ORDER FOR ALL)

1 Definitely true
2 Probably true
3 Probably false
4 Definitely false
8 Or are you not sure
9 (DO NOT READ) Refused

CORO3. Taking vitamin C can prevent a person from being infected with the coronavirus.
Do you believe this is (READ LIST)?

(ROTATE 1-4/4-1 IN THE SAME ORDER FOR ALL)
1 Definitely true
2 Probably true
3 Probably false
4 Definitely false
8 Or are you not sure
9 (DO NOT READ) Refused

CORO4. The coronavirus was created by the Chinese government as a biological weapon.
Do you believe this is (READ LIST)?

(ROTATE 1-4/4-1 IN THE SAME ORDER FOR ALL)
1 Definitely true
2 Probably true
3 Probably false
4 Definitely false
8 Or are you not sure
9 (DO NOT READ) Refused

CORO5. The ways to prevent infection with the coronavirus include regular hand washing and avoiding those showing symptoms of respiratory (RES-PER-ATORY) illness.
Do you believe this is (READ LIST)?

(ROTATE 1-4/4-1 IN THE SAME ORDER FOR ALL)
1 Definitely true
2 Probably true
3 Probably false
4 Definitely false
8 Or are you not sure
9 (DO NOT READ) Refused

CORO6. Some in the U.S. Centers for Disease Control and Prevention, also known as the C-D-C, are exaggerating the danger posed by the coronavirus in order to damage the Trump presidency.
Do you believe this is (READ LIST)?

(ROTATE 1-4/4-1 IN THE SAME ORDER FOR ALL)
1 Definitely true
2 Probably true
3 Probably false
4 Definitely false
8 Or are you not sure
9 (DO NOT READ) Refused

CORO7. Some in the U.S. Centers for Disease Control and Prevention, also known as the C-D-C, are exaggerating the extent of the opioid (O-PEA-OYD) epidemic and its consequences in order to further a political agenda. Do you believe this is (READ LIST)?

(ROTATE 1-4/4-1 IN THE SAME ORDER FOR ALL)

1 Definitely true
2 Probably true
3 Probably false
4 Definitely false
8 Or are you not sure
9 (DO NOT READ) Refused

(ROTATE VERBAIGE IN PARENS)

CORO8. If one person gets the (seasonal flu) and another gets the (coronavirus), which person do you think is more likely to die from the disease: (READ LIST)

(ROTATE IN SAME ORDER AS QUESTION)

1 The person with seasonal flu
2 The person with coronavirus
3 or they are equally likely to die of the disease they have
4 (DO NOT READ) Depends
8 (DO NOT READ) Don’t know
9 (DO NOT READ) Refused

CORO11. There are a number of threats America faces today that might affect the quality of our lives. Please indicate how much you agree with the following statements that compare the challenges of the coronavirus the country faces now to that of other risks the country faces now. (INSERT ITEM). Do you: (READ LIST)?

(SHOW FOR ITEMS B-D)
(INSERT ITEM). Do you: (READ LIST)?

1 Strongly agree
2 Agree
3 Somewhat agree
4 Somewhat disagree
5 Disagree
6 Strongly disagree
9 (DO NOT READ) Refused

(DO NOT ROTATE)

a. I feel the current coronavirus epidemic poses a greater threat to my future quality of life than does the threat of immigration from Mexico.
b. I feel the current coronavirus epidemic poses a greater threat to my future quality of life than does the threat of large natural disasters.
c. I feel the current coronavirus epidemic poses a greater threat to my future quality of life than does the threat of terrorism.
d. I feel the current coronavirus epidemic poses a greater threat to my future quality of life than does the threat of global warming.

COR09 [PN: SHOW FOR FIRST ITEM ONLY]: How much information do you get from each of the following sources?

[PN: SHOW FOR FIRST ITEM ONLY]: Use a scale from 0 to 5, where 0 means you get “NO information” from these sources, and 5 means you get “A LOT of information” from these sources. Of course, you can use any number between 0 and 5. How much INFORMATION do you get from (INSERT ITEM)?

[PN: SHOW FOR SECOND ITEM ONLY]: How much INFORMATION do you get from (INSERT ITEM)? Use a scale from 0 to 5, where 0 means you get “NO information” from these sources, and 5 means you get “A LOT of information” from these sources. Of course, you can use any number between 0 and 5.

[PN: SHOW FOR THE REMAINING ITEMS]: How much INFORMATION do you get from (INSERT ITEM)?

[PN: SHOW FOR THE REMAINING ITEMS]: (IF NECESSARY: Use a scale from 0 to 5, where 0 means you get “NO information” from these sources, and 5 means you get “A LOT of information” from these sources. Of course, you can use any number between 0 and 5.)

0 0- No information
1 1
2 2
3 3
4 4
5 5 – A lot of information
8 (DO NOT READ) Don’t know
9 (DO NOT READ) Refused

(SCRAMBLE ROTATE)

a. Sources such as Fox News, Rush Limbaugh (Lim-BAH), Breitbart (Bright – bart) News, One America News or The Drudge Report
b. Sources such as MSNBC, Bill Maher (MAR), or Huffington Post
c. Sources such as ABC News, CBS News, or NBC News
d. Sources such as Google News or Yahoo News
e. Sources such as Facebook, Twitter, or YouTube
f. Sources such as Associated Press, The New York Times, the Washington Post, or the Wall Street Journal
